# Supplementary material for: High-Performance Photoresistors Based on Perovskite Thin Film with a High PbI2 Doping Level
Source: Nanomaterials (Basel). 2019 Apr 1;9(4):505. doi: 10.3390/nano9040505 (PMC6523679; doi:10.3390/nano9040505)
Supplement: Supplementary file 1 [file nanomaterials-09-00505-s001.pdf]

Supplementary Information

High-Performance Photoresistors Based on Perovskite Thin Film with a High PbI_2 Doping Level

Jieni Li [1], Henan Li [2,*], Dong Ding [1], Zibo Li [1], Fuming Chen [3], Ye Wang [4], Shiwei Liu [1], Huizhen Yao [1], Lai Liu [1] and Yumeng Shi [1,5]

1 International Collaborative Laboratory of 2D Materials for Optoelectronics Science and Technology of Ministry of Education, College of Optoelectronic Engineering, Shenzhen University, Shenzhen 518060, China; jnli91@szu.edu.cn (J.L.); ddinjlu@hotmail.com (D.D.); lizibo@szu.edu.cn (Z.L.); shiweidanielliu@gmail.com (S.L.); yaohz@szu.edu.cn (H.Y.); liu229019@163.com (L.L.); yumeng.shi@szu.edu.cn (Y.S.)

2 College of Electronic Science and Technology, Shenzhen University, Shenzhen 518060, China

3 School of Physics and Telecommunication Engineering, South China Normal University, Guangzhou 510006, China; fmchen@m.scnu.edu.cn

4 Key Laboratory of Material Physics of Ministry of Education, School of Physics and Engineering, Zhengzhou University, Zhengzhou 450052, China; wa0001ye@e.ntu.edu.sg

5 Engineering Technology Research Center for 2D Material Information Function Devices and Systems of Guangdong Province, College of Optoelectronic Engineering, Shenzhen University, Shenzhen 518060, China

* Correspondence: henan.li@szu.edu.cn; Tel.: +86-755-86576530

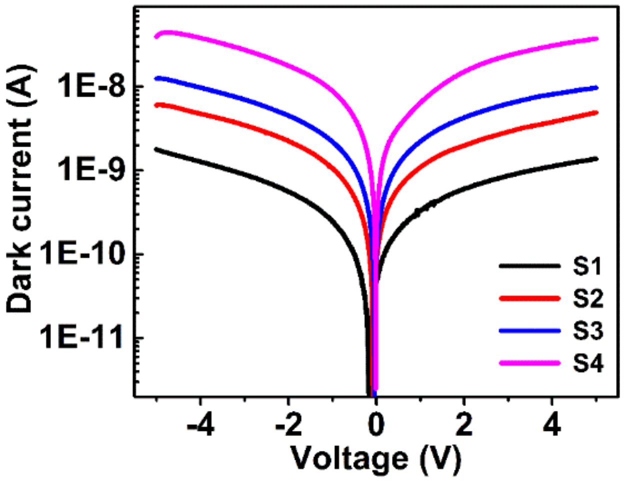

Figure S1. The dark I-V curves of all the devices fabricated from perovskite films with different MAI: PbI_2 ratio.

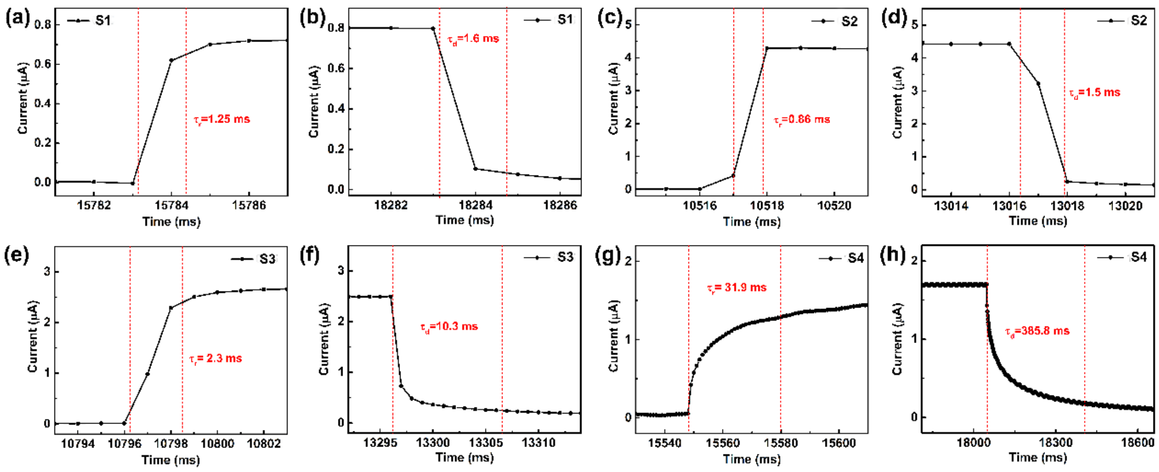

Figure S2. The enlarged rise edges and decay edges of all the devices under white LED light illumination of 4.5 mW/cm^2 at 5 V bias.

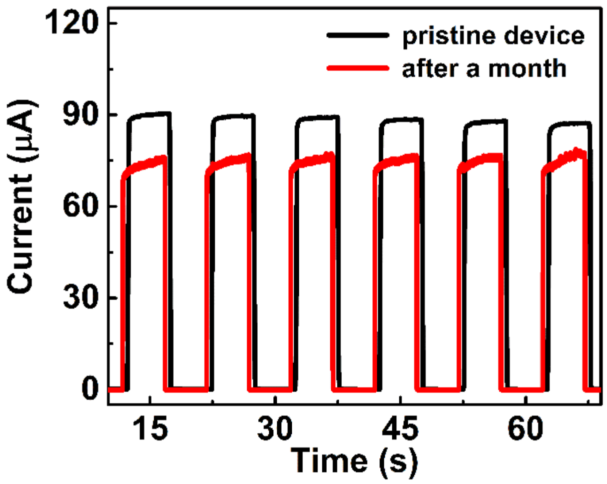

Figure S3. The I-t curves of the device measured as pristine and after a month under 100 mW/cm^2 light illumination.
